# A re‐usable 3D‐printed training model for the endodontic management of dental trauma in immature teeth

**DOI:** 10.1111/iej.14235

**Published:** 2025-04-10

**Authors:** Eva Maier, Jan Zentgraf, Eveline Angele, José Zorzin, Michael Taschner, Matthias Widbiller, Kerstin Galler, Thomas Schratzenstaller

**Affiliations:** ^1^ Department of Operative Dentistry and Periodontology University Hospital Erlangen Erlangen Germany; ^2^ Laboratory for Medical Devices, Department of Mechanical Engineering University of Applied Sciences Regensburg Regensburg Germany; ^3^ Regensburg Center of Health Sciences and Technology (RCHST) University of Applied Sciences Regensburg Regensburg Germany; ^4^ Regensburg Center of Biomedical Engineering (RCBE) University of Applied Sciences Regensburg Regensburg Germany; ^5^ Department of Conservative Dentistry and Periodontology University Hospital Regensburg Regensburg Germany

**Keywords:** 3D‐printing, dental education, dental traumatology, immature teeth, revitalization, training model

## Abstract

**Aim:**

To develop a 3D‐printed model enabling treatment simulation of trauma‐related endodontic and restorative procedures like revitalization or placement of an apical plug on teeth with incomplete root formation.

**Methodology:**

To generate a realistic training model suitable for trauma‐related simulations, CT‐Data sets were segmented, combined, adjusted and optimized using computer‐aided design features. Specific focus was on optimally reflecting characteristics of immature teeth like open apical foramina and thin root walls. For reflection of the revitalization process, a set‐up including a tube filled with red liquid under pressure was developed to be perforated during the procedure by the trainee, simulating bleeding from the apical papilla into the root canal system. Design was based on a combination of cost‐effective simple parts combined with 3D‐printed components to achieve maximal accessibility, exchangeability and re‐usability. Model assembly and preparation were described in step‐by‐step instructions.

**Results:**

As a result of the developmental process, the presented model qualifies for the training of the endodontic management of complications on immature teeth after dental trauma. Step‐by‐step descriptions aligned with the clinical procedures are performable for a revitalization procedure, for pulpotomy followed by fragment re‐attachment and splint application and for placement of an apical plug using hydraulic calcium silicate cements.

**Conclusions:**

The model successfully achieved its intended objectives, so that it may be used in the future in various dental trauma treatment simulations for undergraduate and postgraduate education on the endodontic management of trauma‐related therapies in immature teeth. Its common manufacturing process, affordability and re‐usability support accessibility and sustainability.

## INTRODUCTION

Emergency as well as ongoing treatment of teeth that suffered a traumatic injury is of crucial importance for their long‐term success in aesthetic and functional clinical sense (Alani et al., 2023). The correct treatment plan can prevent complications leading to growth inhibition and infraposition, especially in children and adolescents with incomplete root formations or outstanding growth of surrounding tissues like the alveolar bone and soft tissues (Galler et al., 2021). If vital pulp therapy is not successful, no further root growth is to be expected, and subsequent endodontic management of the respective teeth may present major challenges (Dobroś et al., 2023).

As a theoretical background, guidelines for the management of traumatic dental injuries are available on a national and international level (Bourguignon et al., 2020; Day et al., 2020; DGMKG and DGZMK, 2022; Fouad et al., 2020). Additionally, web‐based information and applications for mobile phones were introduced to simplify receiving information on demand (Andreasen et al., 2012; Duruk & Gümüşboğa, 2022). Nevertheless, developing profound practical skills to handle a clinically demanding emergency with young patients in pain and accompanying stressed parents aside requires repetitive hands‐on training (Gallichan et al., 2023).

In dental education, students acquire theoretical knowledge and basic practical skills for patient treatments (Wimalarathna et al., 2021). Advanced skills in a complicated clinical setting require further clinical experience and continuing personal development training for dentists (Cvijic et al., 2024). Usually, the treatment of traumatic dental injuries can neither be practised during undergraduate training nor in dental practise in the clinical setting, as they occur irregularly and are very diverse in their presentation. Consequently, training on models might present the most universal option to exercise those treatment scenarios in a purposeful manner.

Moreover, innovative treatment options can emerge, necessitating further education of dental practitioners. An example is the revitalization for immature teeth as a modern regenerative endodontic treatment approach. The procedure has developed from case reports to randomized clinical trials finally resulting in clinical recommendations by specialist societies (Galler et al., 2016). This biological approach with the aim of stimulating the growth of pulp‐like tissue into the root canal was translated into a training model solely for this purpose by Widbiller et al. (2018). In this set‐up, a mock blood containing a coagulating substance was incorporated into a reservoir on the top of the root of an immature tooth replica in a gypsum model, enabling training of the clinical procedure of inducing bleeding into the root canal and covering the developed blood clot.

Modern production processes like milling and 3D‐printing enable digital workflows, as well as quick and easy reproduction of exchangeable parts. Concerning resource, financial savings and accuracy, 3D‐printing offers significant advantages over subtractive technologies, which furthermore generate a considerable amount of waste (Barazanchi et al., 2017; Dawood et al., 2015; Javaid & Haleem, 2019). The term' 3D printing' is used collectively to describe a range of additive manufacturing processes, each of which exhibits significant differences in resolution, cost, mechanical properties and optical characteristics, as well as a diverse range of available materials. Fused filament fabrication (FFF), which is one of the most accessible techniques, lacks in surface quality, whilst the powder‐based methods such as selective laser sintering (SLS) are slow and cost‐intensive with an increased need for safety precautions (Damanhuri et al., 2021; Ngo et al., 2018). As stereolitography (SLA) is easy to handle, uses a wide range of materials, has high resolution and reasonable mechanical properties of the printed parts, it is the 3D‐printing technique which is most common for dental applications (Dostalova et al., 2018; Etemad‐Shahidi et al., 2020).

Many examples show the successful use of 3D‐printed models of the oral cavity, which are already in use for various dental applications, showing good results in imitating mechanical and haptical properties of hard tissues, but shortcomings in reflecting soft tissue behaviour, for example in the training of surgical procedures (Dobroś et al., 2023; Höhne et al., 2024). Multiple studies showed that those models are well accepted by students for hands‐on training (Hu et al., 2023; Reymus et al., 2019; Richter et al., 2022). Concerning dental traumatology in the context of 3D‐printed applications, training models with different foci have been developed (Reymus et al., 2018; Zafar et al., 2020). Zafar et al. presented a simplified model for training of replantation and splinting of an avulsed central incisor (Zafar et al., 2020). By segmenting CBCT data of a 16‐year‐old boy after trauma, Reymus et al. developed a patient‐specific model to practise emergency treatment of a variety of dental traumas on mature upper incisors like avulsion, lateral luxation, vertical root fracture and a complex crown fracture (Reymus et al., 2018). Both studies showed that besides efficient economical production processes, the applications were considered highly realistic and helpful for hands‐on training (Reymus et al., 2018; Zafar et al., 2020). A recent publication describes a model where structural changes for immature teeth are manually created rather than printed (Hu et al., 2025). However, to the authors' knowledge, there is currently no available 3D‐printed training model on dental traumatology that focuses on the treatment of immature teeth.

Therefore, the aim of this study was to develop a 3D‐printed model containing teeth with incomplete root formation to enable practising trauma‐related endodontic and restorative procedures like revitalization or placement of an apical plug in a reproducible training situation.

## MATERIALS AND METHODS

### Segmentation of CT‐data

**FIGURE 1:**
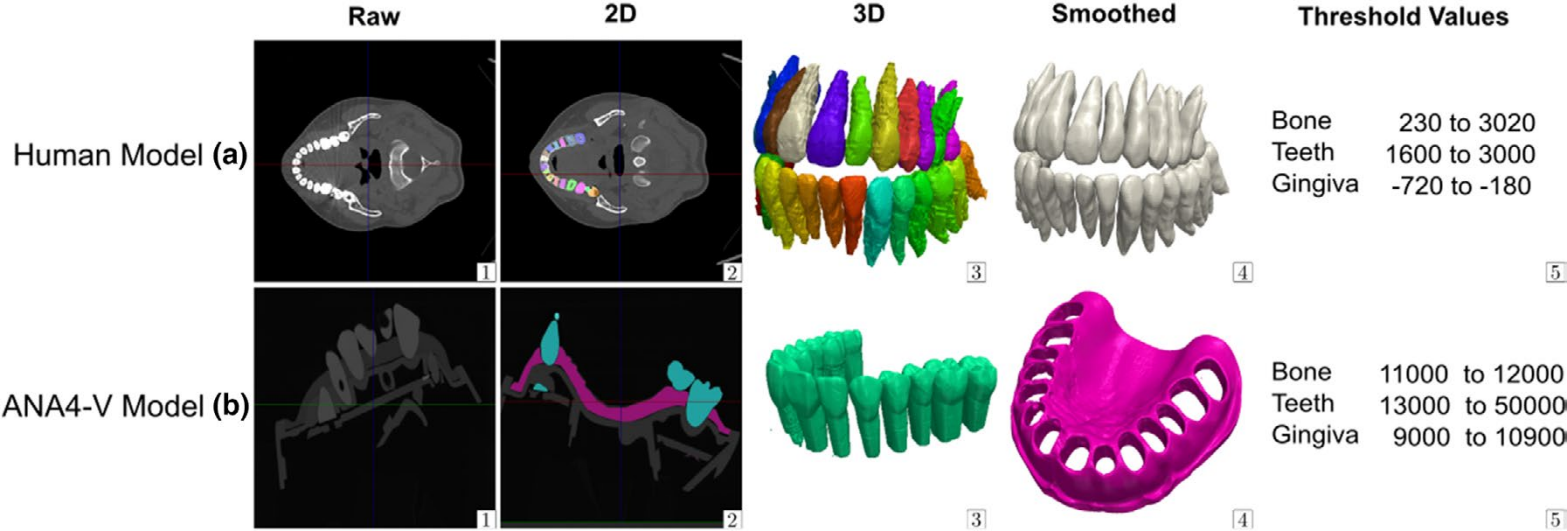
Process of segmentation of CT datasets: Upper row shows segmentation of human CT (a) Lower row of training model μ‐CT (b); (1) Raw 2D data, (2) Shows 2D‐segmentation, (3) Unfiltered 3D Segmentation, (4a) Human teeth after filtering with recursive Gaussian, (4b) Gingiva mask of training model after filtering with recursive Gaussian; (5) Used threshold ranges for segmentation of bone, teeth and gingiva in Simpleware ScanIP Software.

For generating a realistic dental root model, a CT scan of a 32‐year‐old female patient, which was taken because of a mid‐face fracture, was employed (slice thickness: 1 mm). To avoid implementing functional wear and restorative applications specific to the patient in the model situation, a validated training model with standardized teeth and occlusal surfaces was utilized and subjected to a second CT scan. In addition, the gingival model mask was also able to provide an optimal surface template. The tooth roots were generated from the CT scan of the human jaw, whilst the occlusal surfaces were obtained by segmenting the second CT scan, which is a μ‐CT dataset of an existing training model (frasaco GmbH, ANA‐4 V, Tettnang, Deutschland) (Figure 1). A μ‐CT (Baker Hughes Digital Solutions GmbH, phoenix v|tome|xs 240/180, Celle, Deutschland) was used to generate the CT dataset of the training model. Simpleware ScanIP™ (Synopsys Inc., Mountain View, CA, USA) was used as segmentation software. Both datasets were exported into separate STL files and combined subsequently.

### Computer‐aided design (CAD) process

**FIGURE 2:**
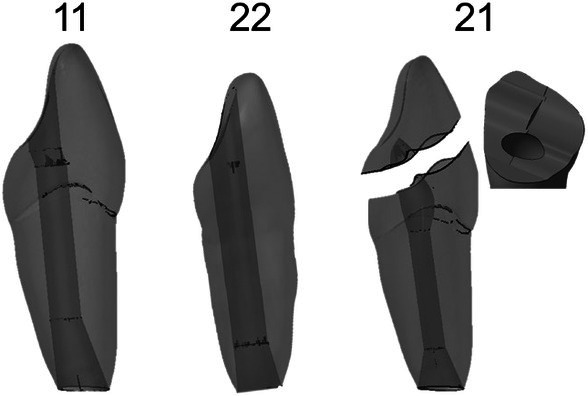
Final design of teeth 21, 11 and 22. 21 has a detail on the fractured surface showing the included pulp chamber; Root apex and access cavity are visible.

Further editing of the exported STL files was performed with Autodesk Netfabb (Autodesk, Inc., version M‐2017.06, San Rafael, CA, USA). Uniting the tooth crown with the high‐detail occlusal surfaces and the anatomical roots was the first boolean operation that was performed. The resulting body was then smoothed by the Software Spaceclaim (ANSYS, Inc., version 2022 R1, Canonsburg, PA, USA) so that the unification area was invisible. The upper jaw was selected as the site for this procedure, specifically the teeth 11, 21 and 22, as this area is most often affected by dental trauma. A total of three anterior teeth (11, 12, 13) were processed this way, as the others were mirrored. To implement an immature root anatomy (Cvek class 3 situation [Cvek 1992]), modifications were necessary on the root canal systems, mainly width of the root canal, thickness of the root walls and anatomy of the apical foramen (blunderbuss). For more straightforward data editing, the generated STL files were converted into STEP files by Software SolidWorks (Dassault Systèmes SolidWorks Corporation, version 2022, Waltham, MA, USA) and imported into CAD Software SolidEdge (Siemens, version 2022, München, Deutschland) for these design modifications. Besides the root anatomy modification, a simulated trepanation for tooth 11 and 22 was added (Figure 2). Tooth 21 was intended for exercises including tooth fractures with a pulp involvement requiring pulpotomy. Subsequently, the pulp chamber was reconstructed in accordance with the anatomical conditions, and an artificial fracture was generated (Figure 2). In the case of molar teeth, only slight modification to geometry was added to ensure a proper and tight fit. This modification included a pin connection containing a hole in the molars and a pin in the alveolar bone of the upper jaw.

As bleeding in the root canal is essential during the revitalization process, the bleeding scenario should be simulated in the model. Therefore, a heat‐shrinkable tube (Stecker Express GmbH, item no.:136306, Reutlingen, Germany) containing artificial blood was placed directly at the root tip. Perforation of the tube through the root canal initiated intracanal bleeding simulation. To implement this technically, a hole to the maxillary frame and the gingiva mask were added, which created a watertight seal between the maxillary frame, tooth 11 and the heat‐shrinkable tube. The 3.2 mm diameter fitted accurately as the pre‐shrunk heat‐shrinkable tubes were insertable and tight enough to guarantee function. These operations were performed with Netfabb software. A fixation was added to revitalization training tooth 11. To ensure watertightness by correct positioning, a cut out for an M2 nut (DIN EN ISO 4032) was designed which could be used to fix tooth 11 with a M2 × 10 headless screw (DIN EN ISO 4027) to the maxillary frame. The surface of the gingival mask was smoothed (Netfabb) and the hole for fixation of the headless screw was added.

As the model was intended to be used in two ways (a stand‐alone model and also incorporated into a phantom head), an attachment option to the phantom head was added. Therefore, a locking plate was designed (SolidEdge) which contained an M6 nut (DIN EN ISO 4032) and could be screwed to the upper jaw frame. The M6 nut also fit with the phantom head screw, allowing it to be mounted onto the phantom head.

### 3D‐printing process

For model fabrication, two different process technologies and two different printers were used. The gingival mask, all teeth and the maxillary frame were produced by Formlabs Form3 (Formlabs Inc., early 2019 version, Somerville, MA, USA) pre‐processing and slicing the STL files with formlabs slicing software PreForm (Formlabs Inc., version 3.24.1, Somerville, MA, USA). As the teeth and the maxillary frame should be stiff and strong enough to withstand subsequent treatment simulations, a rigid resin (Formlabs Inc., White V4, Somerville, MA, USA.) was used (slice thickness: 0.05 mm). In contrast, for the gingival mask, an elastic resin (Formlabs Inc., Elastic 80a, Somerville, MA, USA) was used, which was coloured by the Formlabs soft tissue kit (slice thickness: 0.1 mm). One print job per material produced all necessary parts for one model. After removing the print jobs from the print plate, they were washed with FormWash (Formlabs Inc., Somerville, MA, USA) in TPM (Tripropylenglykolmonomethylether, Formlabs Inc., Somerville, MA, USA) for 20 minutes, and additionally, the small cavities in teeth and the maxillary frame were rinsed with a TPM‐filled syringe. After rinsing the parts under fresh water, they were postprocessed in a Formlabs oven (Formlabs Inc., FormCure, Somerville, MA, USA): the rigid resin parts (teeth and maxillary frame) for 30 min at 60°C, and the elastic resin material for 10 min at 60°C. Both settings were default settings for the respective material and necessary to fully polymerize the materials. The final step involved the removal of all support structures and their residues on the surface of the parts at the tooth roots to obtain a smooth surface that fitted perfectly in the maxillary frame cavities. The removal of these residues was carried out manually by a grinding device (DREMEL Europe, Model 4000, Breda, The Netherlands). The base plate of the maxillary frame was printed with a FFF (Fused Filament Fabrication) printer (Prusa Research a.s, MK3s+, Prague, Czech Republic) and PETG Filament (Fiberlab S.A., Fiberlogy PETG red, Brzezie, Poland). To avoid the issue of support removal and because the resolution of the base plate did not warrant the use of a manufacturing technique with higher resolution, the more economical FFF printing was utilized. Prusaslicer software (Prusa Research a.s, version 2.4.2, Prague, Czech Republic) was used to generate the manufacturing data (g‐code). Finally, an M6 nut was glued to the base plate (Pattex Ultra gel, Henkel AG & Co. KGaA, Düsseldorf, Germany), and an M3 thread was cut in the mounting hole of the maxillary base to combine the maxillary base and base plate by screws (M3x10, DIN EN ISO 4762).

### Model preparation

Prior to the insertion of all teeth into their designated location in the maxillary frame, tooth 22 and the lower fragment of tooth 21 were prepared for the apical plug placement and pulpotomy exercise. This preparation involved the placement of a red‐dyed foam pellet in the tooth, simulating the remaining healthy dental pulp and the freshly wounded tooth (tooth 21), as well as the periapical tissue at the base of the alveolus (lower fragment of tooth 22).

**FIGURE 3:**
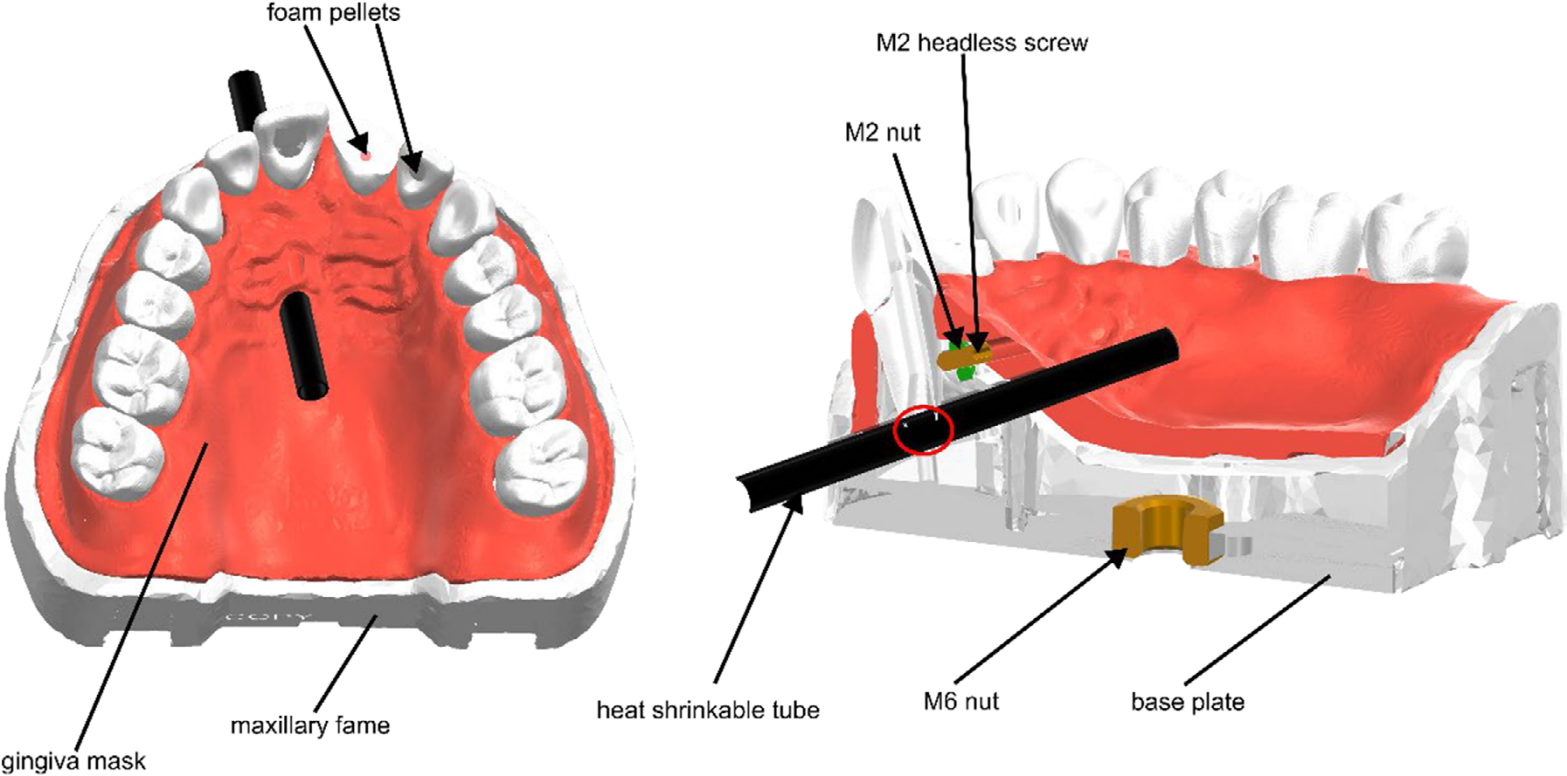
Rendered image of the designed 3D model. Full view on the left, sectional view along revitalization tooth 11 on the right. Arrows describe the individual parts to be used, the lines indicate the general model components. Red circle shows region where tooth presses on heat‐shrinkable tube for watertight fit.

**FIGURE 4:**
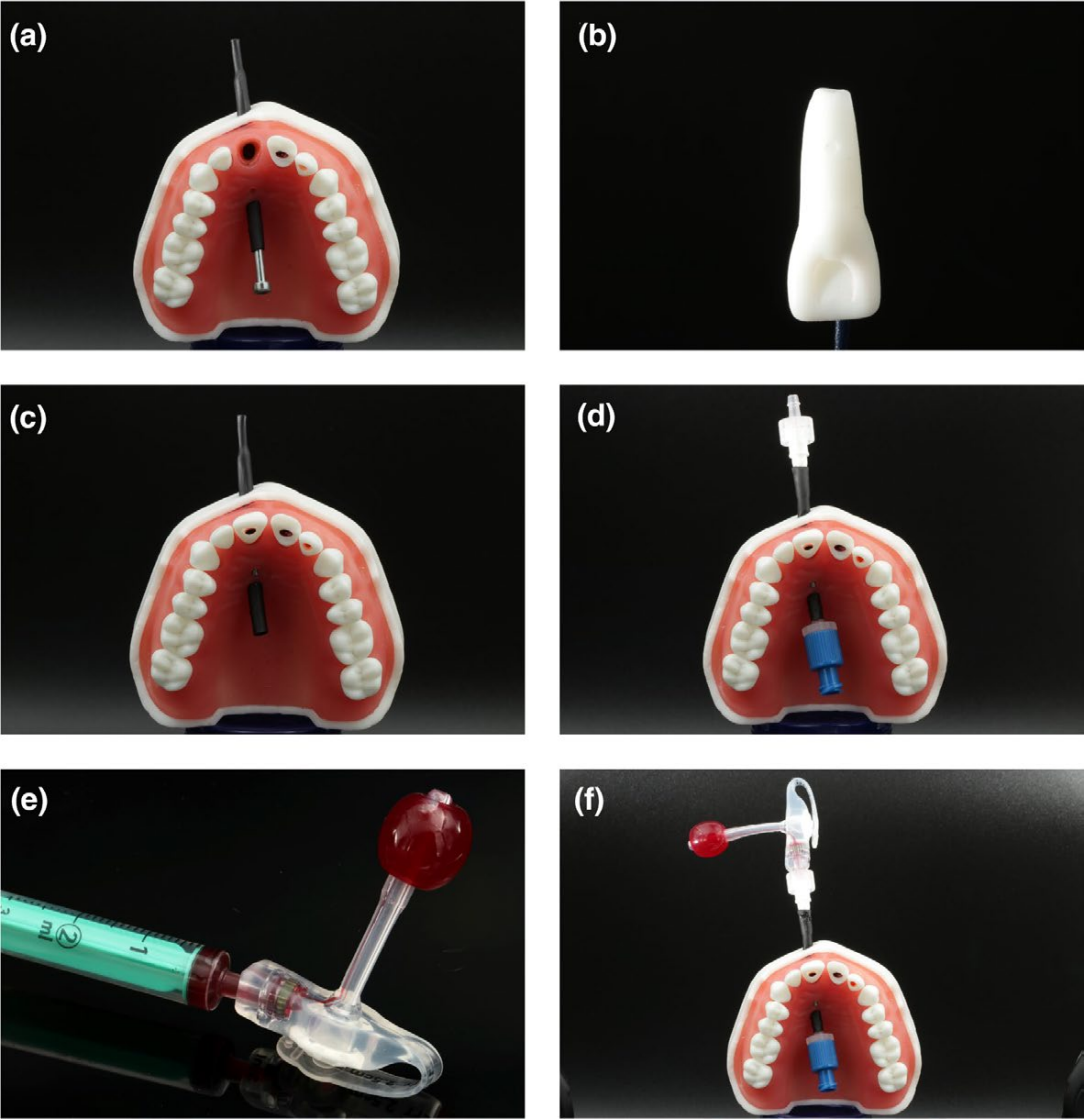
Model assembly for simulating the revitalization of a tooth with immature root development. (a) Insertion of the heat‐shrinkable tube assisted by an M3 x 35 mm cylinder head screw. (b) Upper central right incisor exhibiting immature root development. (c) Model with inserted central right incisor secured by the palatal screw. (d) Heat‐shrinkable tube with a cannula connector attached to the vestibular side and a Luer lock connector linked to the palatal end. (e) Safety balloon of a low‐profile gastrostomy feeding tube filled with ~2 mL of simulated blood. (f) Assembled model for revitalization with the safety balloon of the gastrostomy tube connected to the tube.

For the simulation of revitalization, the model underwent further preparation. Initially, a 40 mm long heat‐shrinkable tube was pre‐treated in an oven (Memmert GmbH + Co. KG, UF160 Plus, Schwabach, Germany) at 120°C for 10 min. This process caused the tube to shrink to a suitable size, ensuring that the design was sufficiently tight to avoid leakage but could still be easily inserted into the cut‐out (Figure 3). Following this, a screw (M3 × 50 mm, DIN EN ISO 4762) was inserted into the tube with the screw acting as a threading aid. The heat‐shrinkable tube, with threading assistance, was passed through the large opening on the palatal side of the model, ensuring that the lengths of the tube ends were equal on the oral or vestibular side (Figure 4a). After threading, the screw was removed from the tube. The central right maxillary incisor, which had an immature root (Figure 4b), was positioned in the alveolus and firmly secured with a screw (Figure 4c). Care was taken to ensure that the apical area of the tooth root was tightly sealed to the heat‐shrinkable tube and verified using loops or a surgical microscope. Two cannula connectors (Microsolv Technology Corporation, Luer connector (male) to size 3/32″, Greater Wilmington, NC, USA) were consecutively firmly attached to the vestibular and palatal ends of the heat‐shrinkable tube. Another luer lock connector (Microsolv Technology Corporation, Luer connector (female) to size 3/32″, Greater Wilmington, NC, USA) was applied to the vestibular end (Figure 4d), and a luer lock closure (Microsolv Technology Corporation, Luer lock closure (female), Greater Wilmington, NC, USA) to the palatal end. To create a blood reservoir, the safety balloon of a low‐profile gastrostomy feeding tube (danumed Medizintechnik GmbH, danuButton® size 12Fr, Regensburg, Germany) was filled with 2 mL of red‐dyed tap water (STAEDTLER 8811‐61 – Noris Club Fingermalfarbe Mali water‐based, Stadler, Germany, Figure 4e). The prepared safety balloon of the gastrostomy tube was attached to the vestibular connector of the heat‐shrinkable tube (Figure 4f).

## RESULTS

With the methodological approach described step‐by‐step, a functioning 3D‐printable, re‐usable model for training of trauma‐related therapeutic procedures was developed. The model allows training in hand as well as in a phantom head simulating the clinical treatment situation.

The model has been designed for re‐usability, with all components (teeth, gingiva mask, maxillary frame, base plate and heat‐shrinkable tube) being either re‐usable or exchangeable. Depending on the applied training and the resulting damage to the components, used parts can be replaced individually and easily with new components. In the case of the revitalization exercise, solely tooth 11 and the heat‐shrinkable tube need to be replaced to create a new training opportunity. The material costs of the resin utilized in the production of a tooth are estimated to be ~0.30€ (161€ per litre), with the cost of a piece of the heat‐shrinkable tube amounting to around 0.03€ (10€ per 15.8 m).

### Simulating revitalization (of a tooth with incomplete root development)

**FIGURE 5:**
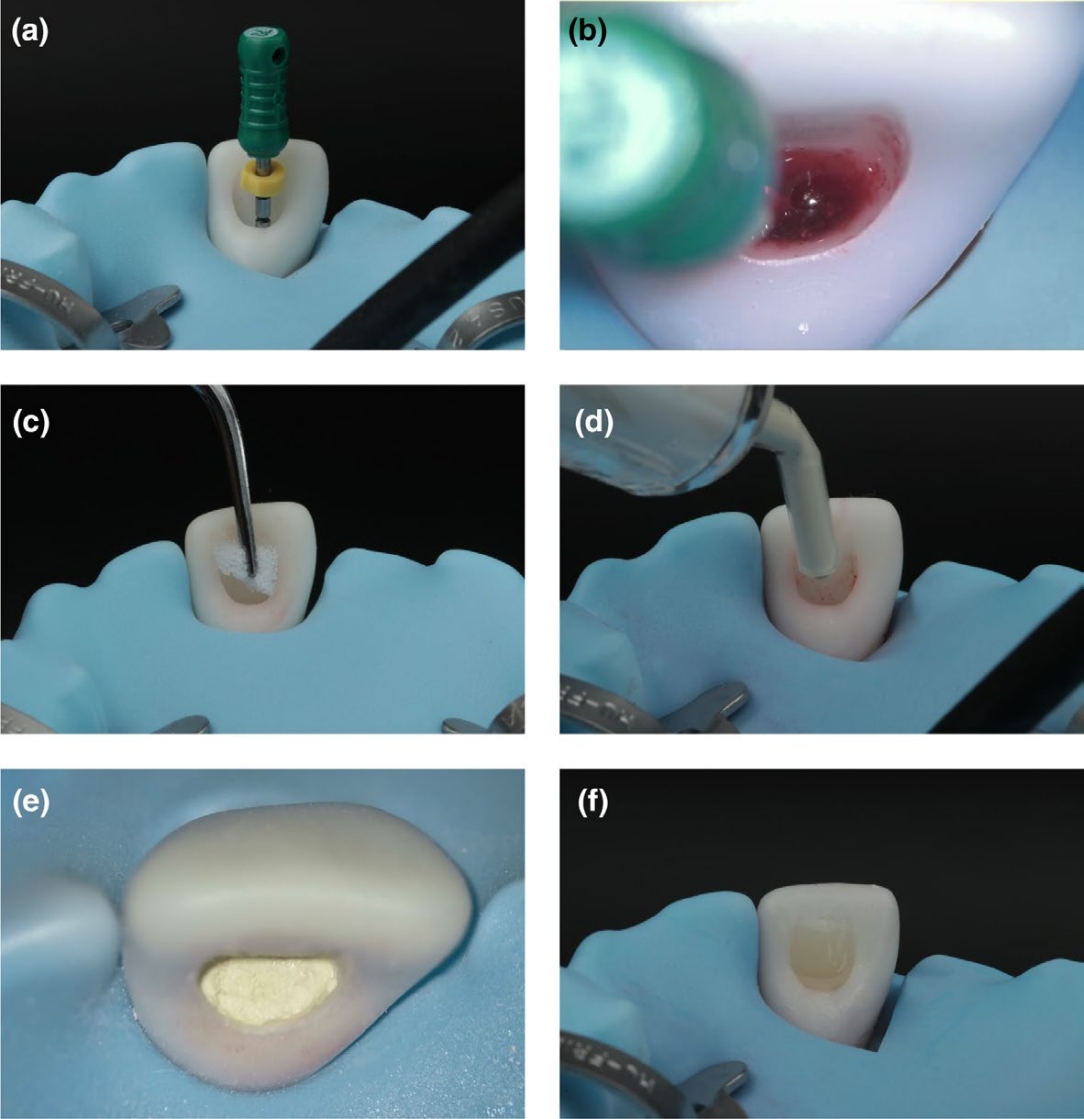
Simulation of the revitalization procedure of a tooth with incomplete root development. (a) Instrumentation with a hand file to induce bleeding by perforating the tube. (b) Simulated blood clot in the root canal under magnification with an operating microscope (this results in colour and white balance differences to macroscopic images). (c) Coverage of the blood clot with a matrix. (d) Application of hydraulic calcium‐silicate cement. (e) Coronally sealed root canal system after setting of the hydraulic calcium‐silicate cement under magnification with an operating microscope (this results in colour and white balance differences to macroscopic images). (f) Direct adhesive filling of the access cavity.

As described above, the model was prepared for revitalization of the upper right central incisor. For a more realistic situation, a rubber dam should be placed. To simulate treatment, the root canal system can be irrigated as recommended by respective guidelines and dried with paper points. Subsequently, simulated bleeding from the apical papilla was induced by pinching a small hole into the heat‐shrinkable tube using a large hand file in a picking and rotating motion (Figure 5a) until bleeding occurred (Figure 5b). The resulting blood clot was covered with a sterile collagen sponge, which may be imitated with a simple foam pellet for cost reasons (Figure 5c). The pellet supported the consecutively applied hydraulic calcium‐silicate cement (Figure 5d). When the hydraulic calcium silicate cement material was set (Figure 5e), the cavity could be restored with an adhesively attached resin‐based composite material (Figure 5f).

### Simulating pulpotomy and fragment re‐attachment

**FIGURE 6:**
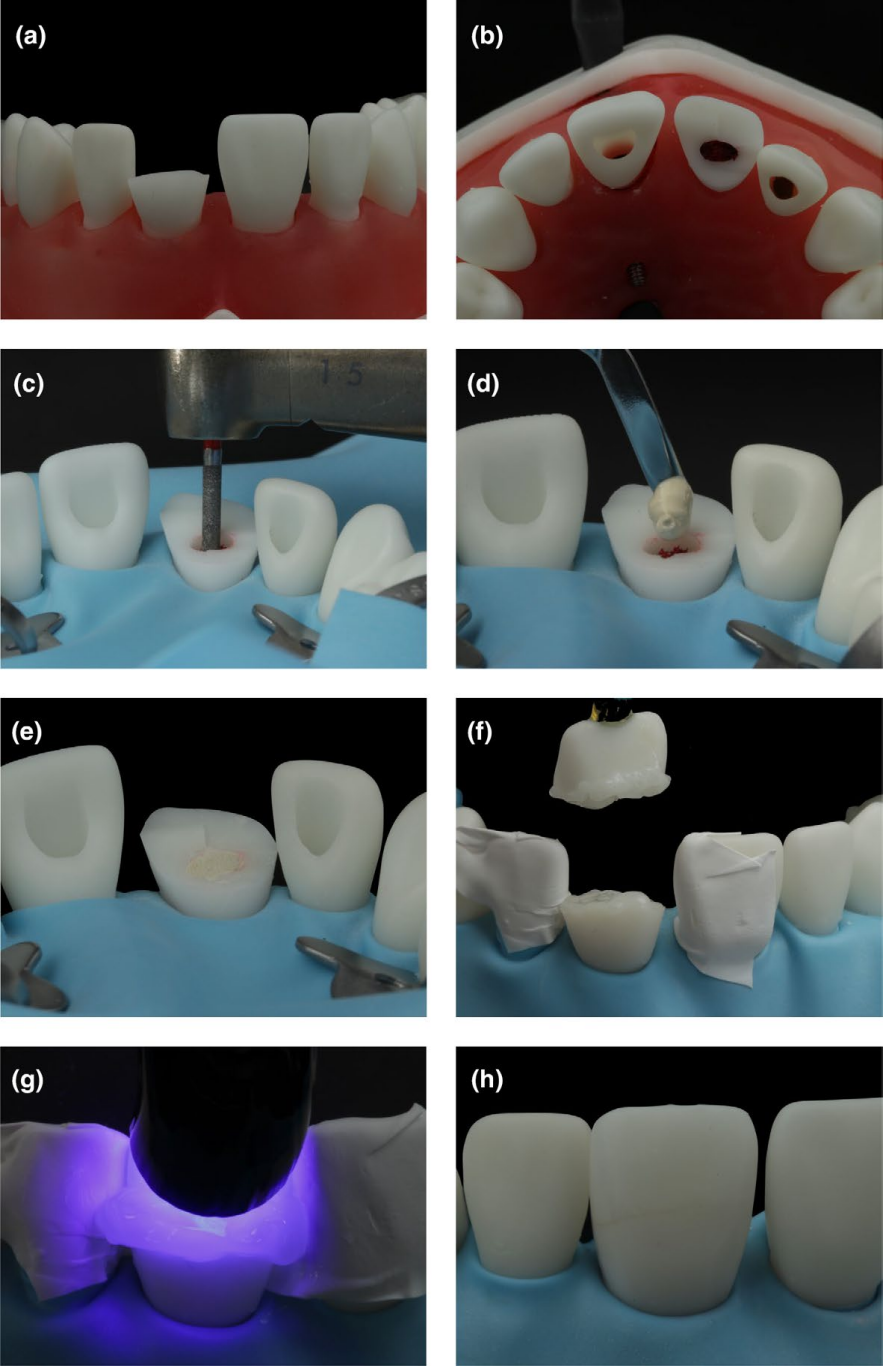
Simulating pulpotomy and fragment re‐attachment. (a) Complicated enamel‐dentin fracture with pulp exposure. (b) Red foam pellet placed in the tooth to simulate the remaining healthy dental pulp tissue. (c) Removal of the coronal 2 mm of the simulated pulp with a diamond bur. (d) Application of hydraulic calcium‐silicate cement. (e) Set hydraulic calcium‐silicate cement. (f) Adhesively pre‐treated tooth fragment attached to a “pick and stick” ready for re‐attachment. (g) Light curing of the resin composite is done under air exclusion using glycerin gel. (h) Restored tooth with the re‐attached fragment after polishing.

Complicated tooth fractures are common injuries and affect enamel, dentin and the dental pulp tissue. A pulpotomy followed by restoration or fragment re‐attachment (if still available) is recommended to maintain the vitality of the remaining pulp tissue. In the presented model, the upper left central incisor suffered a complicated enamel‐dentin fracture involving the pulpal tissue, as indicated by the open pulp chamber (Figure 6a,b). The red‐dyed foam pellet simulated the remaining healthy dental pulp and the freshly wounded tooth (Figure 6b). For training procedures, the coronal 2 mm of the simulated pulp were removed with a diamond bur under water cooling (Figure 6c). Clinically, direct pressure or NaOCl (1%–3%) was applied to the tissue to stop bleeding. Although the model will not achieve haemostasis, this step can still be simulated for training. Afterwards, a hydraulic calcium‐silicate cement was applied (Figure 6d). Once the material had set (Figure 6e), the tooth and the loose fragment were prepared (selective enamel etching with 35%–40% phosphoric acid) and re‐attached in the original position using adhesive and a flowable resin‐based composite material (Figures 6f) and light‐cured under air block gel (Figure 6g). Subsequently, any residual fixation material was removed with a scaler, and the adhesive area could be polished with rubber polishing points and discs, resulting in the restored tooth (Figure 6h).

As an alternative to fragment re‐attachment, any type of direct or indirect restoration can be trained, for example, the model provides various opportunities for teaching aesthetic restorations using direct resin‐based composite materials, ranging from simple procedures like a palatal silicone mould to more elaborate techniques like injection moulding.

### Simulating application of a dental trauma splint

**FIGURE 7:**
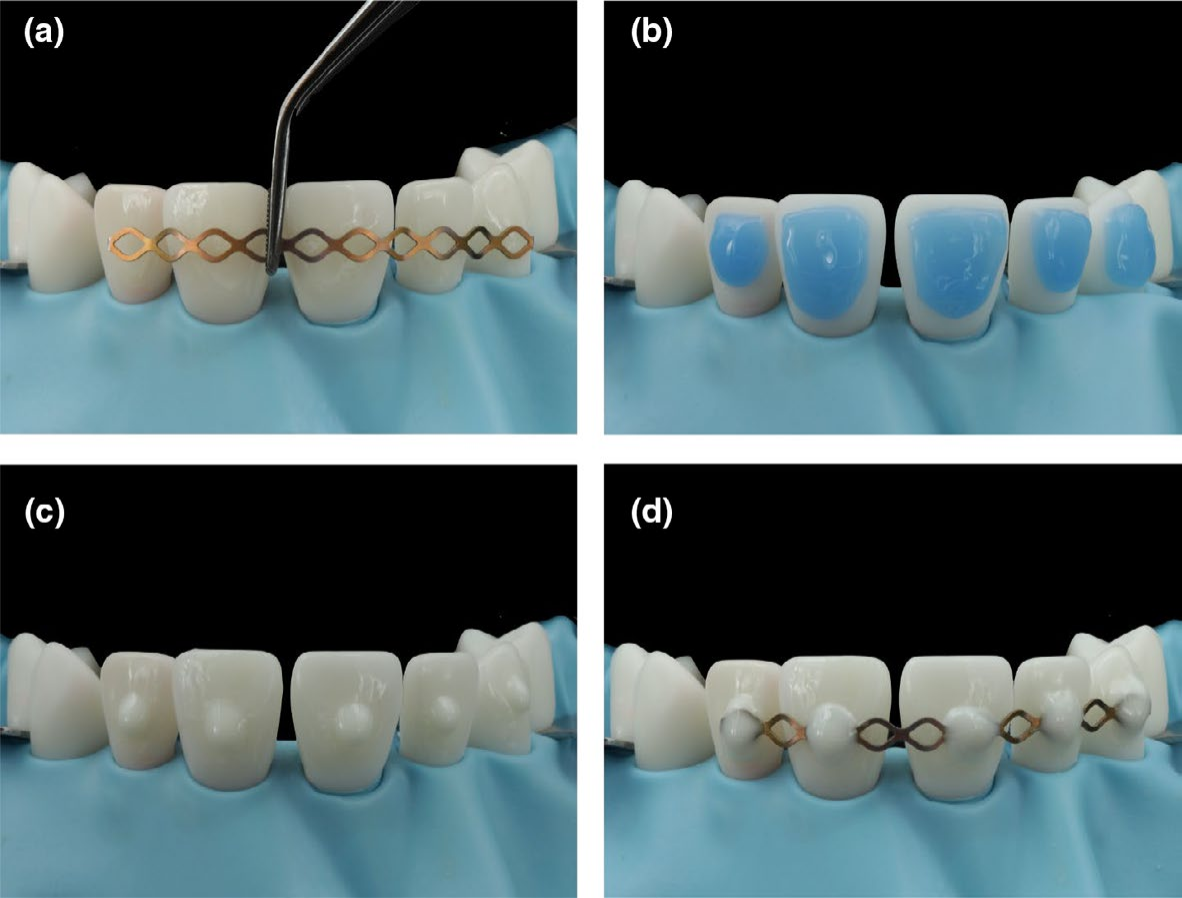
Simulating the application of a dental trauma splint on enamel. (a) Semi‐rigid trauma splint after adjusting its length and shape. (b) Enamel etching using phosphoric acid gel. (c) Flowable resin composite dots placed on the etched teeth. (d) Final result of the dental trauma splint after light curing before removing rubber dam.

In addition to tooth fractures, luxations can also occur and the injuries often happen side by side or even simultaneously. Luxation injuries require immediate repositioning of the tooth back to its original position, if displaced, and consecutive fixation using a splint. In particular, the splinting of the injured tooth was optimally simulated on the model. At first, the splint was adjusted in length and shape using scissors and pliers (Figure 7a). The areas where the splint was adhesively luted to the tooth must be pre‐treated according to the tooth substrate present. Enamel was etched for at least 30 seconds with 35%–40% phosphoric acid gel (Figure 7b) and thoroughly rinsed off with air–water spray. In case of ceramic restorations as an adhesion substrate, sandblasting of the ceramic with alumina at low pressure (e.g., 35 microns grain size and 1.0–1.5 bar pressure) would be applied and followed by a ceramic primer. To fix the splint, dots of flowable resin were applied to the teeth at specific points. To make it easier to remove the splint after successful treatment, a resin with colour mismatch to the tooth is recommended, for example, white opaque (Figure 7c). The splint was placed onto the flowable composite dots and light‐cured. Additional flowable material was applied to the light‐cured dots to increase the splint's retention and avoid sharp edges (Figure 7d).

### Simulating apical plug placement

**FIGURE 8:**
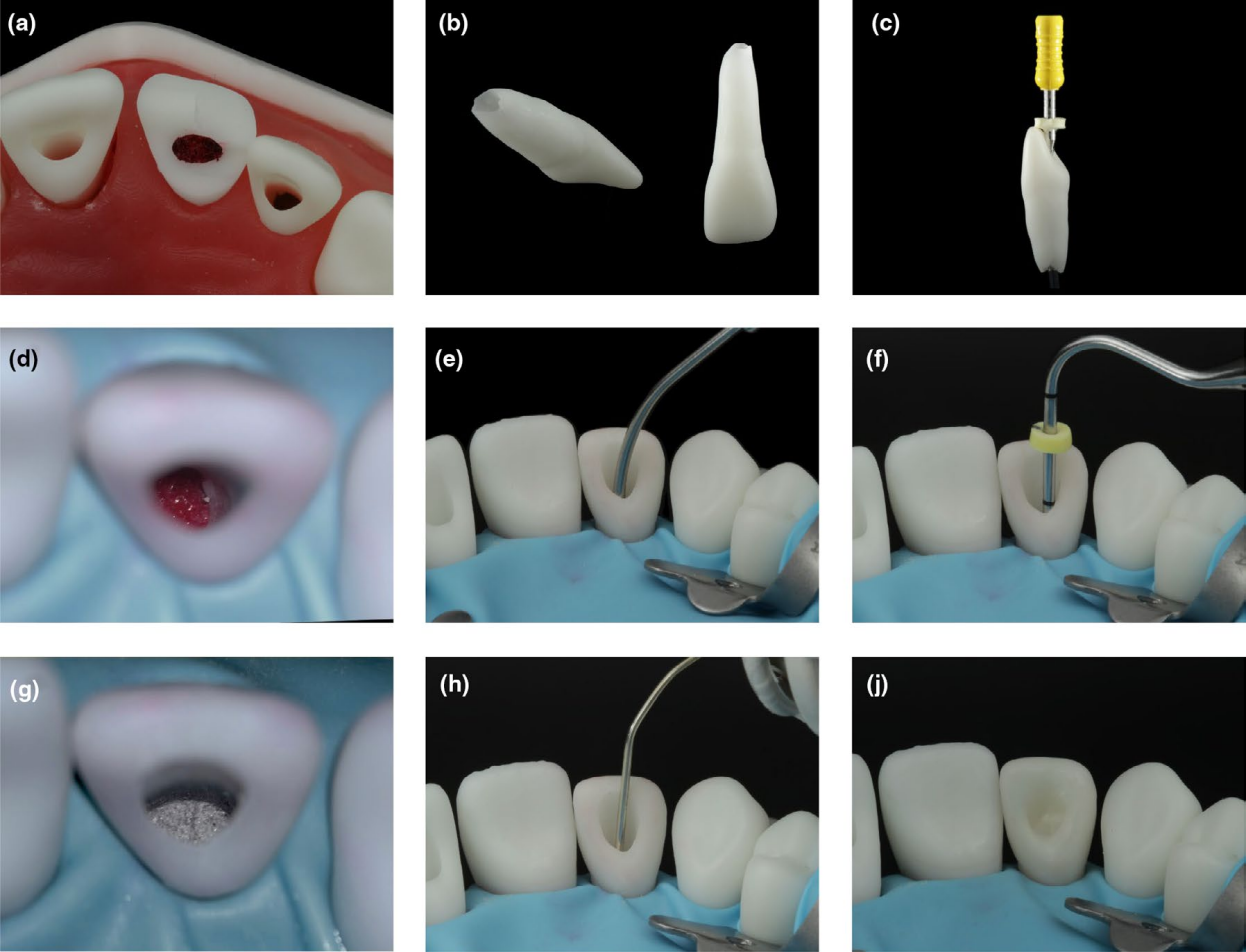
Simulating the application of an apical plug. (a) Simulation model, featuring the upper central right incisor for revitalization, the upper central left incisor for partial pulpotomy, and the upper lateral left incisor for apical plug therapy. (b) Upper lateral left incisor with incomplete root development and wide‐open apical foramen. (c) Extracted upper lateral left incisor with an ISO 50 hand file inserted into the canal up to the apex. (d) Apical region with a red foam pellet simulating the periapical tissue under magnification with an operating microscope (this results in colour and white balance differences to macroscopic images). (e) Application of hydraulic cement with an MTA gun. (f) Condensation of the apical plug with a hand plugger. (g) Apical MTA plug under magnification with an operating microscope (this results in colour and white balance differences to macroscopic images). (h) Backfill of the root canal with heated gutta‐percha (BeeFill, VDW, Germany). (j) Final adhesive restoration of the access cavity.

Besides revitalization, the application of an apical plug with hydraulic calcium‐silicate cement is a recommended treatment option for immature teeth with pulp necrosis. The model presented features of an upper left lateral incisor (Figure 8a) that exhibited incomplete root growth and a wide‐open apical foramen (Figure 8b) to train this procedure. For treatment simulation, the working length was determined preferably by measuring the training tooth outside the model. A suitable endodontic instrument was inserted into the canal up to the apex (Figure 8c), establishing the measuring length. The working length for the apical plug was then determined and transferred to an appropriate instrument, such as a hand plugger and/or MTA gun. After this procedure, the tooth was securely reinserted into the alveolus. The use of magnification loops with attached lights or a dental microscope is highly recommended for training to first check the red‐dyed foam pellet inserted at the base of the alveolus, simulating the periapical tissues (Figure 8d). Following rinsing and drying of the root canal according to the recommended protocols, various methods of applying the material in the apical area were demonstrated and practised, that are, placing the material with an MTA gun (Figure 8e) and condensing the material using a hand plugger (Figure 8f). After applying the apical plug (Figure 8g), warm vertical obturation techniques with gutta‐percha guns and specific heat‐carrying pluggers were also employed (Figure 8h). To complete the treatment, the access cavity was sealed with an adhesively luted resin‐based composite restoration (Figure 8j).

## DISCUSSION

3D‐printed models have become an integral part of dental training over the last decades and have been used for both under‐ and postgraduate training for dental traumatology. However, their focus was primarily on luxation injuries and teeth with complete root formation (Reymus et al., 2018; Zafar et al., 2020), with limited representation of endodontic management and restorative measures. Thus, the aim of this work was to develop a 3D‐printed model of teeth with incomplete root formation to enhance training in endodontic management of dental trauma. The model addresses the specific challenges associated with treating immature teeth in children and adolescents, providing a valuable tool for improving clinical skills in this demanding area.

The model offers the opportunity to realistically practise complex endodontic and restorative procedures on teeth with incomplete root growth. The first of these is the possibility to practise revitalization protocols for immature teeth as an evolution from a proven gypsum training model (Widbiller et al., 2018). Furthermore, application of an apical plug with endodontic materials as an alternative treatment option for teeth with incomplete root growth can be trained. Beyond that, trauma‐related therapies that are important for immature, but also mature teeth can be practised, such as pulpotomies, tooth fragment re‐attachment and splinting of luxated teeth. Combining multiple options for both dentists and students gives flexibility in individual teaching needs and reduces the need for multiple training models, where only one application can be practised (Hanisch et al., 2020; Zafar et al., 2020).

One limitation of the presented model from a clinical point of view is that no luxation injuries and accordingly no repositioning step can be simulated. Avulsion and subsequent replantation were simulated on a comparable model before; however, due to the lack of soft tissue in the bone socket, the model scenario did not reflect blood coagulation, tissue texture and the controlled force that is needed during the clinical procedure (Plotino et al., 2020; Zafar et al., 2020). Another possible limitation might be the colour and material properties of the teeth and surrounding tissues. With the intention of creating an affordable model that can easily be produced with printers common in dentistry, only single‐material parts were printed that could be assembled consecutively. Multi‐material printers could be able to produce layered objects with different material characteristics, for example teeth with hard tissues and soft pulpal tissues in the centre or tooth sockets that are surrounded by a periodontal‐like soft material to simulate lateral luxation injuries (Maier et al., 2019). From an endodontic point of view, not being able to use an apex locator or radiographs to determine the working length must be mentioned as a shortcoming of the presented model. Those features would increase the complexity of the model but should be considered for subsequent versions and developments to better reflect the actual clinical procedures.

From a construction point of view, the presented prototype used stereolithography (SLA) as the currently most common and therefore most available additive manufacturing process in dentistry (Dostalova et al., 2018; Etemad‐Shahidi et al., 2020). All additional materials that were incorporated in the model are cheap and easy to obtain in a medical setting (e.g. gastrostomy feeding tube) or in a DIY store (e.g. heat‐shrinkable tube, nuts and screws). Compared with other models that directly copied anatomical structures from a CBCT scan (Reymus et al., 2018), the current approach used construction engineering to adjust the information received from clinics to the purpose of the model. This made it possible to eliminate individual anatomical variations and create optimal stabilized conditions for training.

Furthermore, the presented prototype demonstrates notable economic efficiency in addition to the practical benefits as the production and personal costs may be high. In addition, the model is favourable in terms of long‐term use with multiple applications (a revitalization session costs about 0.32€). From a construction perspective, the interchangeability of all components represents a further advantage, allowing new treatment simulations, new traumata implementation and other dental training applications, all of which can be easily applied to the existing model. In terms of any other treatment, simply the respective tooth has to be adjusted. This approach has a further environmental effect, as the re‐usable model does avoid a considerable amount of waste.

Unlike the precursor version of the revitalization model, no blood clot formation was implemented in the current process, which could be rated as a limitation. However, this was chosen on purpose as practising the clinical procedure of inducing a bleeding, covering it with a collagen sponge and consecutively a hydraulic calcium silicate cement worked satisfactorily without simulating coagulation. Implementing a blood clot with the required chemicals would severely compromise the shelf life of the components. Nevertheless, this new set‐up would also allow the option to incorporate the mixture described by Widbiller et al. to fully represent clinical reality if necessary (Widbiller et al., 2018). A further constructional limitation was that the optical appearance of the model was compromised by the protruding heat‐shrinkable tube and the Luer lock connectors. The space underneath the teeth, between the maxillary frame and the base plate, offers significant potential for positioning a revitalization mechanism internally within the model, thus ensuring their invisibility in further model development.

The manufacturing process offers further potential for improvement. The current manufacturing technology (SLA), which is a prevalent method in the dental field (Dostalova et al., 2018; Etemad‐Shahidi et al., 2020), necessitates manual removal of the attachment points of the support structure on the surface using a grinding tool. Whilst it is feasible to optimize the positioning of the support structures, manual reworking of the surface cannot be completely prevented due to the manufacturing method. As described above, it is also important to note that the tooth can only be printed from one material due to the production method, and the inclusion of several mechanical properties would require a multi‐stage manufacturing process. It is therefore advisable to examine the feasibility and benefits of an alternative, multi‐material manufacturing technology, which makes it possible to use different material properties for different anatomical components in one print.

## CONCLUSIONS

The model successfully achieved its intended objectives, demonstrating applicability in various dental trauma treatment simulations, including revitalization, apical plug placement and pulpotomy, whilst maintaining a cost‐effective profile. Its common manufacturing process and affordability make it accessible to a wide audience. Additionally, its re‐usability and low production costs enhance its potential for long‐term use. Consequently, the model presents a viable tool for both undergraduate and postgraduate training in endodontic management of trauma‐related situations in immature teeth.

## AUTHOR CONTRIBUTIONS

**Eva Maier and Jan Zentgraf:** Conceptualization, data curation, formal analysis, funding acquisition, investigation, methodology, project administration, software, validation, visualization, writing – original draft preparation. **Eveline Angele:** Conceptualization, data curation, investigation, methodology, software, writing – original draft preparation. **José Zorzin:** Data curation, validation, visualization, writing – original draft preparation. **Michael Taschner:** Conceptualization, resources, validation, writing – review andand editing. **Matthias Widbiller:** Conceptualization, methodology, validation, writing – review andand editing. **Kerstin Galler:** Conceptualization, methodology, project administration, resources, supervision, writing – review and editing. **Thomas Schratzenstaller:** Conceptualization, funding acquisition, methodology, project administration, resources, software, supervision, writing – review and editing.

## FUNDING INFORMATION

This research was funded by Regensburg Centre of Health Sciences and Technology (RCHST) and Regensburg Centre of Biomedical Engineering (RCBE). E.M. was supported by the Interdisciplinary Centre for Clinical Research (IZKF) at the University Hospital of the Friedrich‐Alexander University of Erlangen‐Nuremberg (*Junior Project J103*).

## CONFLICT OF INTEREST STATEMENT

The authors declare that they have no conflict of interest concerning the work presented.

## ETHICS STATEMENT

The CT dataset (Material and Methods, Segmentation of CT data) was generated for a patient after suffering a mid‐facial fracture, and was therefore justified due to this medical condition. The CT dataset was consequently available before the project started and not taken specifically for this study.

## Data Availability

The data that support the findings of this study are available from the corresponding author upon reasonable request.
